# The Effect of Timing of Mandibular Distraction Osteogenesis on Weight Velocity in Infants Affected by Severe Robin Sequence

**DOI:** 10.3390/children9030319

**Published:** 2022-02-28

**Authors:** Zhe Mao, Ricardo Battaglino, Jiawei Zhou, Yingqiu Cui, Mayank Shrivastava, Gabriel Tian, Faezeh Sahebdel, Liang Ye

**Affiliations:** 1 Department of Oral and Maxillofacial Surgery, Guangzhou Women and Children’s Medical Center, Guangzhou 510623, China; mzgzwacmc@163.com (Z.M.); zjwgzwacmc@163.com (J.Z.); gzhtwang@163.com (Y.C.); tiangzwacmc@163.com (G.T.); 2 Department of Rehabilitation Medicine, Medical School, University of Minnesota, Minneapolis, MN 55455, USA; rbattagl@umn.edu (R.B.); saheb005@umn.edu (F.S.); 3 Department of Diagnostic and Biological Sciences, School of Dentistry, University of Minnesota, Minneapolis, MN 55455, USA; shriv072@umn.edu

**Keywords:** weight velocity, infant, Robin Sequence, mandibular distraction osteogenesis

## Abstract

Background: Impaired weight gain is prevalent in Robin Sequence (RS) newborns. Although mandibular distraction osteogenesis (MDO) has been proven to improve oral feeding, its impact on postoperative weight gain remains unclear. The purpose of this study is to explore whether MDO can help RS babies reach a normal weight, as well as the effect of MDO timing on weight velocity. Methods: One hundred infants with severe RS and one hundred with normal controls met the inclusion criteria for the study. Included patients underwent MDO. Weights at different timing points were recorded and analyzed and compared to normal controls. Results: After the distractor removal weights of patients undergoing MDO at <1 month and 1–2 months were close to the normal control (6.81 ± 0.93 kg versus 7.18 ± 0.61 kg, *p* = 0.012, and 6.82 ± 0.98 kg versus 7.37 ± 0.75 kg, *p* = 0.033, respectively), the weights of patients undergoing MDO at 2–3 months and 3–4 months still lagged behind (7.56 ± 1.29 kg versus 8.20 ± 0.61 kg, *p* = 0.000206 and 7.36 ± 1.05 kg versus 8.25 ± 0.77 kg, *p* = 0.004, respectively). The weights of all RS infants undergoing MDO showed no significant difference compared to the controls when they aged to 1 year (9.34 ± 0.99 kg versus 9.55 ± 0.45 kg, *p* = 0.254 for MDO at <1 month; 9.12 ± 0.91 kg versus 9.33 ± 0.46 kg, *p* = 0.100 for MDO at 1 to 2 months; 9.38 ± 0.29 kg versus 9.83 ± 0.53 kg, *p* = 0.098 for MDO at 2 to 3 months; and 9.38 ± 0.29 kg versus 9.83 ± 0.53 kg, *p* = 0.098 for MDO at 3 to 4 months). Conclusion: The MDO procedure helped patients with severe RS to reach a normal weight; and MDO intervention was recommended at an early stage for early weight gain.

## 1. Introduction

Robin Sequence (RS) is described as a craniofacial abnormality that demonstrates clinical features of micrognathia, glossoptosis, and upper airway obstruction. These clinical features are frequently associated with U and V shaped clefts in the palate. As congenital malformations which can be characterized into isolated and syndromic presentation, RS has common syndromes that occur in conjunction with Stickler syndrome and velocardiofacial syndrome. The overall estimated incidence of RS is 1:8000 to 1:14,000 [1,2], with several genetic abnormalities having been identified in the etiology of this disorder. It is noticed that isolated RS is associated with SOX9 and Potassium Inwardly Rectifying Channel Subfamily J Member 2 (KCNJ 2) dysregulation on chromosome 17 while syndromic Stickler are associated with alterations in connective tissue collagen genes and velocardiofacial from a microdeletion of 22q 11.2 [3,4].

In infants with RS, micrognathia is a striking feature which along with retrogenia and glossoptosis leads to airway obstruction followed by respiratory distress and feeding impairment. The mechanism of feeding impairment is suggested to be caused by anatomical and developmental alterations [5]. It is observed that in RS, mandibular position is altered causing a physical obstruction of nasal and oral passages by not allowing the tongue base to descend from nasopharynx and by inhibiting the fusion of palate. These alterations limit the formation of negative intraoral pressure as well as negative intrathoracic pressure which successively increases respiratory effort and energy expenditure in infants, causing infants to fail to gain weight in the early post-natal period [5,6].

To address feeding issues and respiratory distress in infants with RS, various methods have been used. These methods can be non-surgical and surgical. The non-surgical methods such as nasopharyngeal tubes, modified palatal plate, and prolonged intubation with supplemental oxygen have shown improvements in weight gain and air way obstruction. However, these measures are proven less effective in infants with moderate and severe respiratory distress or where immediate weight gain is required [7]. In such cases, surgical interventions such as tongue–lip adhesion, MDO, and tracheostomy are used. Due to recent advancements in surgical devices, MDO has become a compelling method in the management of airway obstruction. In general, MDO increases the mandibular length by moving in forward direction as well as simultaneously pulling the tongue base anteriorly. Thus, MDO improves micrognathia as well as glossoptosis, it thereby relives the obstruction and facilitates feeding. Literature has documented the effectiveness of MDO in relieving obstruction and improving oral feeding [8,9,10]. However, previous studies have used small patient cohorts, and varied outcomes of weight gain have been reported [11,12,13,14]. To date, it is still unclear whether MDO helps in improving weight gain in infants with RS. Moreover, it was found that a substantial proportion of the variation in birth weight is due to genetic differences between newborns. Therefore, we should ignore the effect of genetic differences on an individual’s early growth. With regard to infants affected by RS, which is a genetic condition, few studies took birth weight into account. Instead, they focused on the amount of weight those patients gained (weight gain). They ignored whether MDO could correct weight abnormality that may result from genetic difference. Thus, unfortunately, no reported study could be found to confirm whether MDO would help RS babies reach a normal weight, which is what clinicians care about most. This study determines whether MDO can help RS infants reach a normal weight, as well as the effect of MDO timing on their weight velocity. Specifically, we compared weight among RS infants with MDO intervention to normal controls for a subsequent period of time. We hypothesized that MDO could help RS neonates in achieving a normal weight, providing crucial clinical evidence for MDO efficacy in addressing pediatric weight gain abnormalities in RS patients. We also hypothesized the timing of MDO operation matters.

## 2. Materials and Methods

### 2.1. Study Design and Subjects

This is a prospective cohort study of infants with RS who were managed by MDO at the Department of Oral and Maxillofacial Surgery in Guangzhou Women and Children’s Medical Center. The study was approved by the Ethics Committee at Guangzhou Women and Children’s Medical Center. The study population comprised of 100 RS patients and 100 normal controls. The subjects were recruited from 23 November 2016 to 2 September 2021. Written informed consent was obtained from parents or other guardians prior to enrollment. All subjects who had a clinical diagnosis of RS were based on the clinical consensus report of two clinical specialists [15]. Dr. Zhe Mao and Dr. Liang Ye were present at the same time for the diagnosis of any single case. Patients with micrognathia, glossoptosis, and airway obstruction established the initial diagnosis of RS [15,16]. A fibreoptic nasopharyngoscopy examination was conducted in all patients to further confirm glossoptosis and airway obstruction, resulting in the final diagnosis. The patients with breathing or feeding difficulties were considered to have severe RS [15,16]. If they had severe cardiopulmonary disease, head and neck tumors, or trauma leading to changes in the local anatomical structure, laryngomalacia, brain-induced central apnea, or mixed apnea other anomalies causing airway obstruction, subjects were excluded. In all 100 patients with severe RS, MDO was performed as described previously [17,18]. The patient underwent mandibular distraction at a speed of 1.2 mm per day 48 h after surgery. Distraction was performed until the upper and lower jaws were symmetrically aligned or a slight underbite was achieved. No infection or hardware failure was detected in the recruited patients.

### 2.2. Study Variables

Predictor variables included MDO intervention and age at the time of operation which was <1 month, 1 to 2 months, 2 to 3 months, and 3 to 4 months. The primary outcome variable was birth weight, weight at MDO surgery, post-discharge weight, weight at MDO removal, and weight at aged one year. Weight was used as an outcome measure between infants with severe RS and in the normal controls.

### 2.3. Statistical Analysis

A multiple independent t-test was performed to analyze weight statistically among patients with RS and in the normal controls at different time points. The level of significance was *p* < 0.05 (*p* < 0.01: highly significant).

## 3. Results

### 3.1. Sample Distribution

The total study sample included 100 infants with RS and 100 normal controls. The characteristics of patient with RS at different surgical ages, <1 month, 1 to 2 months, 2 to 3 months, and 3 to 4 months are summarized in Table 1. Among the different age groups, 58% were male and 42% were female, overall isolated PRS and syndromic distribution was 81% and 19%, respectively, and associated cleft palate was found in 95% of patients. In this study, there were more cases with cleft palate in each group compared to those without cleft palate. Also, there were more cases affected by isolated RS compared to those affected by syndromic RS.

### 3.2. Weight Outcomes at Different Timing Points

Weight comparisons between RS patients undergoing MDO and those in the normal controls were depicted in Table 2. The birth weight of RS patients was 3.06 ± 0.48 kg, while in the normal control it was 3.26 ± 0.05 kg (*p* = 0.000). We could see these 100 RS patients had a lower birth weight on the whole compared to the controls. The weight difference was 6.13%. At the timing point of MDO intervention, the weight of RS patients was 3.58 ± 0.67 kg, which was still significantly lower than the normal control, which was 4.97 ± 0.91 kg (*p* = 0.000) *t*-score (−16.14). However, the weight difference increased to 38.83%. After the MDO operation and then the removal of distractors, we noticed the weight of RS infants with the value 7.03 ± 0.11 kg was getting close to the normal control with a weight of 7.60 ± 0.83 kg. The weight difference decreased to 7.5%. Finally, as they aged until cleft palate surgery, RS infants showed successful outcomes in weight compared to the normal controls (9.12 ± 0.12 kg versus 9.56 ± 0.09 kg, *p* = 0.01, *t*-score: −3.45) and the difference continued to decrease to 4.60%, which was even smaller than the 6.13% for birth weight.

### 3.3. Weight Outcomes Intervened by MDO Surgical Age

**Figure 1:**
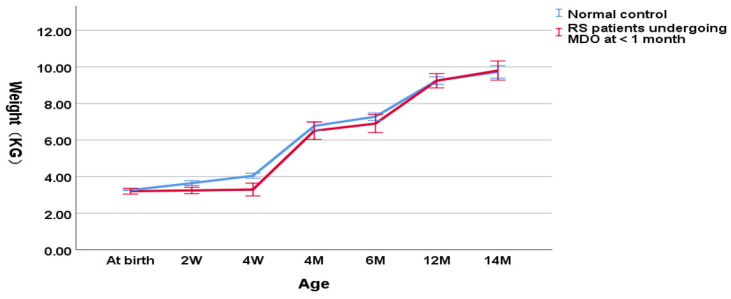
Wight-for-age chart (RS patients undergoing MDO at <1 month versus normal control).

As shown in Figure 1, in the group undergoing MDO at <1 month, the weight of RS patients was decreasing significantly compared to the normal control group (3.29 ± 0.46 kg versus 3.93 ± 0.26 kg, *p* < 0.001 for the weight at the timing point of MDO and 4.03 ± 0.44 kg versus 6.01 ± 1.41 kg, *p* < 0.001 for the post-discharge weight). At the timing point of distractor removal, the weight of RS patients was close to that of the normal controls (6.81 ± 0.93 kg versus 7.18 ± 0.61 kg, *p* = 0.012). At aged one year, patients reached the normal weight compared to the normal controls (9.34 ± 0.99 kg versus 9.55 ± 0.45 kg, *p* = 0.254).

**Figure 2:**
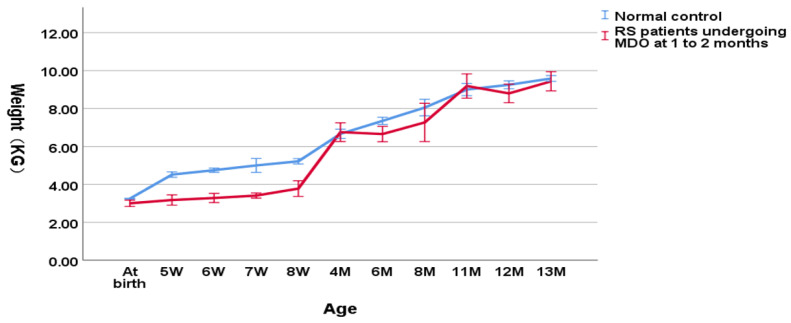
Wight-for-age chart (RS patients undergoing MDO at 1 to 2 months versus normal control).

As shown in Figure 2, in the group undergoing MDO at 1 to 2 months, the weight of RS patients was decreasing significantly compared to the normal controls (3.41 ± 0.50 kg versus 4.80 ± 0.37 kg, *p* < 0.001 for the weight at the timing point of MDO and 3.81 ± 0.58 kg versus 5.36 ± 0.48 kg, *p* < 0.001 for the post-discharge weight). At the timing point of distractor removal, the weight of RS patients was close to the normal controls (6.82 ± 0.98 kg versus 7.37 ± 0.75 kg, *p* = 0.033). At aged one year, the weight of RS patients didn’t show a significant difference compared to the normal controls (9.12 ± 0.91 kg versus 9.33 ± 0.46 kg, *p* = 0.100).

**Figure 3:**
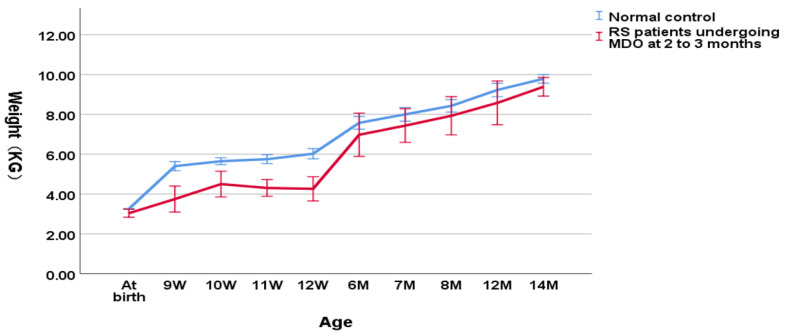
Wight-for-age chart (RS patients undergoing MDO at 2 to 3 months versus normal control).

As shown in Figure 3, in the group undergoing MDO at 2 to 3 months, the weight of RS patients was also significantly lower compared to the normal controls (4.12 ± 0.81 kg versus 5.88 ± 0.33 kg, *p* < 0.001 for the weight at the timing point of MDO and 4.43 ± 0.79 kg versus 6.26 ± 0.46 kg, *p* < 0.001 for the post-discharge weight). However, at the timing point of distractor removal, the weight of RS patients could not reach a normal weight compared to the normal controls (7.56 ± 1.29 kg versus 8.20 ± 0.61 kg, *p* = 0.000206). At aged one year, RS patients finally reached the normal weight compared to the normal controls (9.38 ± 0.29 kg versus 9.83 ± 0.53 kg, *p* = 0.098).

**Figure 4:**
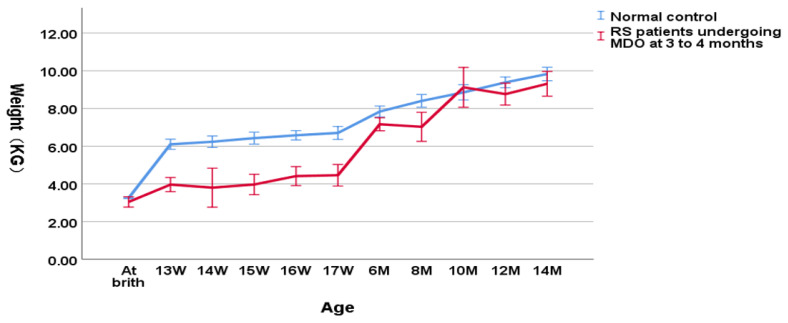
Wight-for-age chart(RS patients undergoing MDO at 3 to 4 months month versus normal control).

As shown in Figure 4, in the group undergoing MDO at 3 to 4 months, the weight of RS patients was still significantly lower relative to the normal controls (3.90 ± 0.79 kg versus 6.31 ± 0.33 kg, *p* < 0.001 for the weight at the timing point of MDO and 4.30 ± 0.79 kg versus 6.63 ± 0.34 kg, *p* < 0.001 for the post-discharge weight). At the timing point of distractor removal, the weight of RS patients could not reach a normal weight compared to the normal controls (7.36 ± 1.05 kg versus 8.25 ± 0.77 kg, *p* = 0.004). At aged one year, RS patients finally reached the normal weight compared to the normal controls (9.38 ± 0.29 kg versus 9.83 ± 0.53 kg, *p* = 0.098).

Therefore, RS patients undergoing MDO at 2 to 3 months and 3 to 4 months continued to lag in weight gain and remained underweight until aged one year compared to the normal controls. However, at the timing point of distractor removal, the weight of RS patients undergoing MDO at <1 month and 1 to 2 months was already close to normal control. An early stage MDO may be recommended for early weight gain in infants affected by severe RS, since early weight gain is very important to the clinical rehabilitation of those patients.

## 4. Discussion

This study demonstrates a consistent weight gain in infants with RS comparable to the non-RS control for a subsequent period of time if MDO intervention was performed at an early stage.

It has been observed that a majority of the patients with RS have difficulty feeding and gaining weight. The possible factors that investigators have identified in previous research led to failure to thrive in infancy such as inadequate calorie intake, malabsorption, and excessive calorie consumption [19]. There can be many reasons for this weight reduction. One of the primary reasons is mechanical airway obstruction due to developmental alterations such as micrognathia, glossoptosis, and cleft palate which have been correlated well with impaired feeding in infants with RS. Similar to RS infants, Down syndrome infants have shown a reduction in growth compared to normal controls [20,21]. However, weight is assessed at different time points in the present study. While in syndromic RS infants other factors, such as anomalies of the base of the skull in Stickler syndrome and nasal constriction, central sleep apnea, and pharyngeal hypotonia in velocardiofacial syndrome with associated cleft palate, exacerbate airway obstruction and impede oral feeding [22]. In addition to these factors, hypoventilation also increases respiratory effort and leads to increased caloric consumption, exacerbating feeding insufficiency [23,24,25,26,27]. Due to these reasons, our study included weight gain in infants with RS as a primary outcome and compared their weight to the normal group after an MDO intervention.

In the literature, various approaches have been reported for delivering additional calories in infants with RS. These methods are gastrotomy tube placement and early feeding by nasogastric tube which allows for early weight gain in RS infants [28]. It is also noted that severe RS infants have reduced sodium levels and supplementation of sodium improved weight gain [29]. However, these methods are likely to succeed in infants with moderate isolated RS, not in severe RS and Syndromic RS infants [30]. For effective management of cases not responsive to conservative approaches, surgical methods such as tongue–lip adhesion, MDO, and tracheostomy can be used. In previous studies, the efficacy of surgical interventions in improving airway obstruction was assessed and MDO was shown to have superior weight gain outcomes compared to other surgical interventions [14,31,32,33]. It is a safe and a viable treatment for feeding impairment in infants diagnosed with RS. Therefore, in the present study we used MDO as an intervention method to assess improvement in weight gain in infants with RS. In a systematic review, analytical investigators observed that infants treated with MDO had an improved ability to feed orally approximately 12 months after surgery [9]. Our study reported similar findings of increased weight gain 12 months after MDO intervention. In our study, compared to the normal controls, RS patients show statistically less weight from birth to the time of the MDO procedure. Later, the weights of some RS patients were close to the normal controls at the time of distractor removal. Finally, RS patients undergoing MDO showed no significant difference in weight compared to the normal control at aged one year. It Improvements in airway obstruction, as well as energy expenditure on breathing, and increased weight gain can be attributed to MDO.

Our findings corroborate the findings of the retrospective study in which the growth of 41 RS infants was assessed. These infants underwent early MDO and showed initial decelerated growth followed by a period of escalation. Similar to our data, the infants in this retrospective study caught up to their healthy peers by aged 12 months [24]. The findings of our study are also similar to findings by Gary et al. where retrospective data was analyzed in 22 infants who underwent MDO. They observed an initial decrease in the growth percentile followed by an increased growth percentile at six months and postoperatively [8]. There are studies which have compared growth curves in PRS infants using MDO and conservative management, and they noticed similar results of improved weight gain following MDO. In a study by Al-Samkari et al., the weights of 18 RS infants managed non-operatively and 12 RS infants managed by operative MDO intervention were assessed. They found that infants treated with MDO, on average, gained 10.9 mg more per day compared to the infants managed non-operatively [28].

In another study, Mudd et al. examined the outcomes of 24 infants with RS. They noted a decrease in growth percentile followed by a gradual increase in growth [12]. Similarly, Khansa et al. prospectively assessed 28 RS patients treated with MDO, tongue–lip, or conservative measures. In the above study, 10 patients treated with MDO reported an average increased growth percentile of 19.5 at 10 to 12 months and 25.3 at 16 to 20 months, respectively [14]. In another study of 73 infants treated with MDO, the patients experienced a declined growth percentile from birth to MDO and significantly increased growth after MDO and postoperatively [11]. Recently, in another review case series, the weight of 24 infants with isolated RS and syndromic RS was compared with the normal controls. In this review study, investigators reported that newborns with and without PRS had similar birth weights, but the growth rate of infants with PRS lagged behind the normal controls even if addressed at an early stage [34].

Although these studies noticed comparable and similar results, none of the studies have so far used such large sample sizes (100 cohorts) and compared the results of weight gain with normal controls. Also, the present study is not specifically designed to analyze the growth curves which were assessed by the previous studies. Our study demonstrates the predictor variables of weight and age at the time of operation. Furthermore, our study demonstrates a consistent weight gain for a subsequent period of time in RS infants if MDO intervention was performed at an early stage, while patients who received MDO at a later stage failed to reach a normal weight compared to the normal controls up to aged one year. Thus, an early MDO intervention needs to be considered in clinical approaches to severe RS patients in order to help those patients with an early weight gain, which had previously gone unrecognized.

In contrast to the present study, there are studies which have reported poor weight-gain following MDO in RS patients [13,35,36]. In a study of 10 RS infants treated with MDO, investigators found that 7 of 10 infants experienced a reduction in growth percentile from the time of MDO and 12 months postoperatively; the infants in the study experienced poor overall weight gain following MDO compared unlike the infants in our research. There are other studies with a small sample size in the literature which reported poor weight gain following MDO. For example, Daniel et al. [36] examined weight gain for 5 infants treated with MDO. This study noted a decline in growth percentile from birth to hospital discharge and an increase from discharge to aged 12 months. The reason for significant differences in these studies is a small sample size as well as a different methodology and selection criteria. Additionally, these studies have limitations that make drawing conclusions regarding weight gain challenging. Our study has a few inherent limitations: data acquisition variation; selection bias in the study and control groups; and variability in surgical technique, complications, and post operative management. Despite these limitations, our patient cohort and long follow up period substantiate the findings which were limited in the earlier investigations.

In conclusion, at birth, RS infants have reduced weight compared to the controls. MDO has a positive influence on the weight of RS infants which helps patients with severe RS to reach a normal weight and the normal weight gain persists until one year post operatively. MDO intervention is recommended at an early stage.

## Figures and Tables

**Table 1:** Characteristics of Patients.

	<1 Month for MDO	1 to 2 Months for MDO	2 to 3 Months for MDO	3 to 4 Months for MDO
Gender (Male/Female)	18/10	19/21	14/3	7/8
Classification (Isolated RS/Syndromic RS)	21/7	31/9	15/2	14/1
Cleft Palate (No/Yes)	0/28	2/38	2/15	1/14
Average Age of Patients Undergoing MDO	2 weeks	6 weeks	11 weeks	14 weeks

MDO: Mandibular Distraction Osteogenesis; RS: Robin Sequence.

**Table 2:** Weight Comparison Between PRS Patients Undergoing MDO and Normal Controls.

Timing Point	Number		Weight (kg)		*t*	*p*
	PRS Patient	Normal Control	PRS Patient	Normal Control		
At birth	100	100	3.06 ± 0.48	3.26 ± 0.05	−4.166	*p* = 0.000066 *p* < 0.01
MDO	100	100	3.58 ± 0.67	4.97 ± 0.91	−16.148	*p* = 0.0000 *p* < 0.01
Post-discharge Weight	100	100	4.05 ± 0.66	5.57 ± 0.76	−17.497	*p* = 0.0000 *p* < 0.01
Distractor Removal	100	100	7.03 ± 0.11	7.60 ± 0.83	−6.407	*p* = 0.0000 *p* < 0.01
Cleft Palate Surgery	100	100	9.12 ± 0.12	9.56 ± 0.09	−3.450	*p* = 0.01

## Data Availability

Not applicable.
